# The American Dental Society of Europe

**Published:** 1901-12

**Authors:** Waldo E. Royce

**Affiliations:** President; 2, Lonsdale Gardens, Tunbridge Wells


### Foreign Correspondence.

### THE AMERICAN DENTAL SOCIETY OF EUROPE.

2, Lonsdale Gardens, Tunbridge Wells,
20th September, 1901.

To the Editor:

Sir,—The members of the American Dental Society of Europe are desirous that a large number of Americans should visit them at the time of their next meeting, and they are especially anxious to have as many of the honorary members present as possible. It is, therefore, with regret that we learn that the date fixed for the National Dental Association meeting is August 5. We do not wish to take people away from that meeting, and we would gladly change the date of our meeting, making it late enough for you to attend both, if it were possible, but you are doubtless aware that our next meeting is to be held in Stockholm. This is a city of rare beauty and is surrounded by places of interest, which afford opportunity for the most delightful excursions. While easy of access, it has been visited by but few. In short, it is an ideal place for holding a meeting in early August, but *it would not be practicable for a late meeting.*

In addition to the attractions mentioned, we have a most capable and energetic local Committee, which is already at work arranging for the entertainment and comfort of visitors.

Last month at their meeting in London both the International Dental Federation and the Association of Advisory Boards adjourned to meet at the same time and place as the American Dental Society of Europe. This gives our meeting an international character, and distinguished members of our profession from all parts of Europe and America have declared that it is their intention to attend.

In fixing the date of our meeting we must therefore think of the convenience of these bodies.

It is usual for the American Dental Society of Europe to hold its meeting the first week in August, at which time Stockholm is at its best. To defer it to the last of the month would undoubtedly detract from the interest of the meeting and very greatly from the pleasure of those attending. Would it not be to the interest of both societies if the meeting at Niagara Falls were changed to some time in July? Then those wishing to attend could do so, and by taking steamer for Hamburg or Bremen could reach Stockholm in time for the meeting of the American Dental Society of Europe, which, if necessary, could be held a little later than usual in order that they might participate. I am assured that if this could be arranged a large contingent would visit us.

I am, therefore, writing to the President and some of the members of the National Dental Association, asking them if they will change the date of their meeting to some time in July, so that those of their members who wish could also attend our meeting.

Those presenting papers at Stockholm will have the advantage of addressing many of the leading members of the profession, as in an international congress; at the same time they will avoid the crowd of a congress and the danger of being obliged to read a paper that has cost many hours of hard work before a small section.

Yours very truly,

WALDO E. ROYCE,

*President.*

